# (*E*)-3-[4-(Hex­yloxy)phen­yl]-1-(4-hydroxy­phen­yl)prop-2-en-1-one

**DOI:** 10.1107/S1600536809019436

**Published:** 2009-05-29

**Authors:** Ibrahim Abdul Razak, Hoong-Kun Fun, Zainab Ngaini, Norashikin Irdawaty Abd Rahman, Hasnain Hussain

**Affiliations:** a X-ray Crystallography Unit, School of Physics, Universiti Sains Malaysia, 11800 USM, Penang, Malaysia; b Department of Chemistry, Faculty of Resource Science and Technology, Universiti Malaysia Sarawak, 94300 Kota Samarahan, Sarawak, Malaysia; c Department of Molecular Biology, Faculty of Resource Science and Technology, Universiti Malaysia Sarawak, 94300 Kota Samarahan, Sarawak, Malaysia

## Abstract

In the title compound, C_21_H_24_O_3_, the enone group adopts an *s*–*cis* conformation. The planes of the aromatic rings are inclined at an angle of 6.1 (1)°. The alk­oxy tail is not linear, with the maximum deviation from the least-squares plane being 0.375 (2) Å. Mol­ecules are connected into extended chains along the *a* axis through O—H⋯O_carbon­yl_ hydrogen bonds and are inter­linked *via* C—H⋯O inter­actions to form a two-dimensional array parallel to the *ab* plane.

## Related literature

For the biological properties of chalcone derivatives, see: Bhat *et al.* (2005 ▶); Xue *et al.* (2004 ▶); Zhao *et al.* (2005 ▶); Satyana­rayana *et al.* (2004 ▶); Won *et al.* (2005 ▶). For related structures, see: Razak *et al.* (2009 ▶); Razak *et al.* (2009*a* ▶,*b* ▶); Ngaini, Fadzillah *et al.* (2009 ▶); Ngaini, Rahman *et al.* (2009 ▶). For the stability of the temperature controller used in the data collection, see: Cosier & Glazer (1986 ▶).
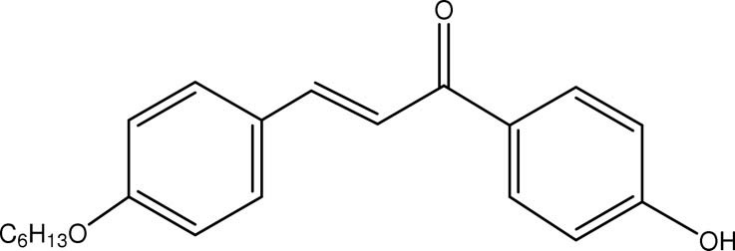


## Experimental

### 

#### Crystal data

C_21_H_24_O_3_
*M* *_r_* = 324.40
Orthorhombic, 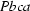

*a* = 10.0237 (2) Å
*b* = 9.7695 (2) Å
*c* = 35.3220 (6) Å
*V* = 3458.96 (12) Å^3^
*Z* = 8
Mo *K*α radiation
μ = 0.08 mm^−1^
*T* = 100 K
0.25 × 0.12 × 0.07 mm

#### Data collection

Bruker SMART APEXII CCD area-detector diffractometer
Absorption correction: multi-scan (*SADABS*; Bruker, 2005 ▶) *T*_min_ = 0.980, *T*_max_ = 0.995
25370 measured reflections
5659 independent reflections
3311 reflections with *I* > 2σ(*I*)
*R*_int_ = 0.086

#### Refinement

*R*[*F* ^2^ > 2σ(*F* ^2^)] = 0.073
*wR*(*F* ^2^) = 0.139
*S* = 1.02
5659 reflections
222 parameters
H atoms treated by a mixture of independent and constrained refinement
Δρ_max_ = 0.27 e Å^−3^
Δρ_min_ = −0.25 e Å^−3^

### 

Data collection: *APEX2* (Bruker, 2005 ▶); cell refinement: *SAINT* (Bruker, 2005 ▶); data reduction: *SAINT*; program(s) used to solve structure: *SHELXTL* (Sheldrick, 2008 ▶); program(s) used to refine structure: *SHELXTL*; molecular graphics: *SHELXTL*; software used to prepare material for publication: *SHELXTL* and *PLATON* (Spek, 2009 ▶).

## Supplementary Material

Crystal structure: contains datablocks global, I. DOI: 10.1107/S1600536809019436/tk2457sup1.cif

Structure factors: contains datablocks I. DOI: 10.1107/S1600536809019436/tk2457Isup2.hkl

Additional supplementary materials:  crystallographic information; 3D view; checkCIF report

## Figures and Tables

**Table 1:** Hydrogen-bond geometry (Å, °)

*D*—H⋯*A*	*D*—H	H⋯*A*	*D*⋯*A*	*D*—H⋯*A*
O1—H1O1⋯O2^i^	0.89 (3)	1.77 (3)	2.6466 (19)	169 (2)
C1—H1*A*⋯O1^ii^	0.93	2.55	3.458 (2)	164

Symmetry codes: (i) 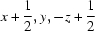; (ii) 
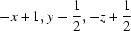
.

**Fig. 1.**
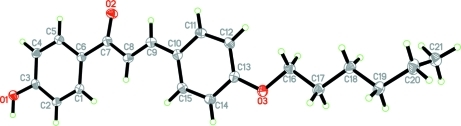
The molecular structure of (I), showing 50% probability displacement ellipsoids and the atom numbering scheme.

**Fig. 2.**
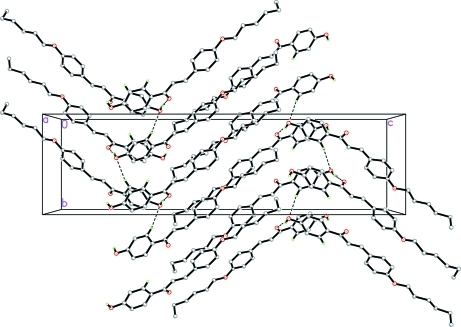
The crystal packing in (I), viewed down the a axis. Intermolecular O—H···O hydrogen bonding and C—H···O contacts are shown as dashed lines. H atoms not involved in hydrogen bondings are omitted for clarity.

## supplementary crystallographic information

### Comment

Chalcones derivatives are reported to demonstrate biological properties such as an anti-malarial (Xue *et al.*, 2004), anti-cancer (Bhat *et al.*, 2005), anti-inflammatory (Won *et al.*, 2005), anti-platelet (Zhao *et al.*, 2005) as well as anti-hyperglycemic (Satyanarayana *et al.*, 2004) activities. Synthetic and naturally occurring chalcones have been extensively studied and developed as pharmaceutically important molecules. As part of our studies, we have synthesized the title chalcone derivative, (I), and tested its anti-bacterial activity against *E. coli* ATCC 8739; the compound showed anti-microbial activity. In this paper, we report the crystal structure of (I).

The conformation of the enone (O2/C7–C9) moiety in (I) is *s*–*cis* with the O2—C7—C8—C9 torsion angle being 5.7 (3)°. The mean plane through the enone moiety makes dihedral angles of 15.9 (1)° and 10.9 (1)°, with the C1–C6 and C10–C15 aromatic rings, respectively. The two aromatic rings form a dihedral angle of 6.1 (1)°.

The short H1A···H8A (2.16 Å) contact resulted in the slight widening of the C1—C6—C7 (123.0 (2)°) and C6—C7—C8 (120.7 (2)°) angles whereas the widening of C8—C9—C10 and C9—C10—C15 angles to 129.0 (2)° and 123.7 (2)° respectively, resulted from the close interatomic contact of H8A···H15A (2.34 Å). Correspondingly, the opening of the O3—C13—C12 (124.9 (2)°) angle is the consequence of strain induced by short H12A···H16A (2.28 Å) and H12A···H16B (2.38 Å) contacts. Similar features can also be found in previously reported related structures (Razak *et al.*, 2009; Razak *et al.*, 2009*a*,*b*; Ngaini, Fadzillah *et al.*, 2009; Ngaini, Rahman *et al.*, 2009).

Even though the C16—O3—C13—C12 torsion angle is 0.8 (3)°, only part of the alkoxyl tail, O3/C16–C18, is co-planar with the attached aromatic ring [maximum deviation of the least-squares plane of O3/C16–C18 is -0.002 (2) Å]. The alkoxyl chain is twisted about the C19—C20 bond as shown by the C18—C19—C20—C21 torsion angle being -73.4 (2)°. The least-squares plane through the the alkoxyl chain, O3/C16–C21, [maximum deviation of 0.375 (2) Å at C16] makes a dihedral angle of 49.1 (2)° with the attached aromatic ring.

In the crystal structure, intermolecular O1—H1O1···O2 hydrogen bonds between the hydroxy and keto groups link the molecules into extended chains along the *a* axis, Table 1. The chains are interlinked *via* C1—H1A···O1 interactions into a 2-D array parallel to the *ab*-plane, Table 1 and Fig. 2.

### Experimental

A mixture of 4-hydroxyacetophenone (1.36 g, 10 mmol), 4-hexyloxybenzaldehyde (2.06 ml, 10 mmol) and KOH (2.02 g, 36 mmol) in methanol (30 ml) was heated at reflux for 24 h. The reaction was cooled to room temperature and acidified with cold diluted HCl (2 N). The resulting precipitate was filtered, washed and dried. After redissolving in a hexane–ethanol (7:1) solution, followed by few days of slow evaporation, crystals were collected.

### Refinement

The O-bound H atom was located in a difference Fourier map and refined freely; O—H = 0.89 (3) Å. All the C-bound H atoms were positioned geometrically and refined using a riding model with C—H = 0.93–0.97 Å. The *U*_iso_ values were constrained to be 1.5*U*_eq_ (methyl-H atoms) and 1.2*U*_eq_ (other H atoms). The rotating model group was applied for the methyl group.

### Crystal data

**Table:**

C_21_H_24_O_3_	*F*(000) = 1392
*M**_r_* = 324.40	*D*_x_ = 1.246 Mg m^−^^3^
Orthorhombic, *P**b**c**a*	Mo *K*α radiation, λ = 0.71073 Å
Hall symbol: -P 2ac 2ab	Cell parameters from 2200 reflections
*a* = 10.0237 (2) Å	θ = 2.3–22.0°
*b* = 9.7695 (2) Å	µ = 0.08 mm^−^^1^
*c* = 35.3220 (6) Å	*T* = 100 K
*V* = 3458.96 (12) Å^3^	Block, colourless
*Z* = 8	0.25 × 0.12 × 0.07 mm

### Data collection

**Table:**

Bruker SMART APEXII CCD area-detector diffractometer	5659 independent reflections
Radiation source: sealed tube	3311 reflections with *I* > 2σ(*I*)
graphite	*R*_int_ = 0.086
φ and ω scans	θ_max_ = 31.3°, θ_min_ = 1.2°
Absorption correction: multi-scan (*SADABS*; Bruker, 2005)	*h* = −14→14
*T*_min_ = 0.980, *T*_max_ = 0.995	*k* = −14→14
25370 measured reflections	*l* = −51→50

### Refinement

**Table:**

Refinement on *F*^2^	Primary atom site location: structure-invariant direct methods
Least-squares matrix: full	Secondary atom site location: difference Fourier map
*R*[*F*^2^ > 2σ(*F*^2^)] = 0.073	Hydrogen site location: inferred from neighbouring sites
*wR*(*F*^2^) = 0.139	H atoms treated by a mixture of independent and constrained refinement
*S* = 1.02	*w* = 1/[σ^2^(*F*_o_^2^) + (0.0389*P*)^2^ + 1.4637*P*] where *P* = (*F*_o_^2^ + 2*F*_c_^2^)/3
5659 reflections	(Δ/σ)_max_ < 0.001
222 parameters	Δρ_max_ = 0.27 e Å^−^^3^
0 restraints	Δρ_min_ = −0.25 e Å^−^^3^

### Special details

**Table:**

Experimental. The crystal was placed in the cold stream of an Oxford Cryosystems Cobra open-flow nitrogen cryostat (Cosier & Glazer, 1986) operating at 100.0 (1) K.
Geometry. All e.s.d.'s (except the e.s.d. in the dihedral angle between two l.s. planes) are estimated using the full covariance matrix. The cell e.s.d.'s are taken into account individually in the estimation of e.s.d.'s in distances, angles and torsion angles; correlations between e.s.d.'s in cell parameters are only used when they are defined by crystal symmetry. An approximate (isotropic) treatment of cell e.s.d.'s is used for estimating e.s.d.'s involving l.s. planes.
Refinement. Refinement of *F*^2^ against ALL reflections. The weighted *R*-factor *wR* and goodness of fit *S* are based on *F*^2^, conventional *R*-factors *R* are based on *F*, with *F* set to zero for negative *F*^2^. The threshold expression of *F*^2^ > σ(*F*^2^) is used only for calculating *R*-factors(gt) *etc*. and is not relevant to the choice of reflections for refinement. *R*-factors based on *F*^2^ are statistically about twice as large as those based on *F*, and *R*- factors based on ALL data will be even larger.

### Fractional atomic coordinates and isotropic or equivalent isotropic displacement parameters (Å^2^)

**Table:**

	*x*	*y*	*z*	*U*_iso_*/*U*_eq_	
O1	0.45711 (14)	0.93154 (14)	0.31262 (3)	0.0203 (3)	
O2	0.17214 (13)	0.81942 (14)	0.15690 (3)	0.0216 (3)	
O3	0.47446 (13)	0.24927 (13)	0.00413 (4)	0.0207 (3)	
C1	0.43756 (18)	0.74282 (19)	0.22313 (5)	0.0177 (4)	
H1A	0.4783	0.6696	0.2109	0.021*	
C2	0.47890 (19)	0.77884 (18)	0.25929 (5)	0.0183 (4)	
H2A	0.5452	0.7286	0.2714	0.022*	
C3	0.42085 (18)	0.89013 (19)	0.27736 (5)	0.0172 (4)	
C4	0.32103 (19)	0.96503 (19)	0.25923 (5)	0.0198 (4)	
H4A	0.2827	1.0402	0.2712	0.024*	
C5	0.27945 (18)	0.92715 (19)	0.22348 (5)	0.0193 (4)	
H5A	0.2127	0.9773	0.2116	0.023*	
C6	0.33582 (18)	0.81466 (18)	0.20480 (5)	0.0163 (4)	
C7	0.28184 (18)	0.77389 (19)	0.16745 (5)	0.0175 (4)	
C8	0.35542 (18)	0.67861 (19)	0.14301 (5)	0.0177 (4)	
H8A	0.4336	0.6379	0.1516	0.021*	
C9	0.30968 (18)	0.65064 (19)	0.10828 (5)	0.0184 (4)	
H9A	0.2357	0.7010	0.1007	0.022*	
C10	0.35965 (18)	0.55225 (18)	0.08071 (5)	0.0169 (4)	
C11	0.29114 (19)	0.5369 (2)	0.04661 (5)	0.0201 (4)	
H11A	0.2197	0.5948	0.0415	0.024*	
C12	0.32554 (19)	0.43872 (19)	0.02008 (5)	0.0201 (4)	
H12A	0.2773	0.4303	−0.0023	0.024*	
C13	0.43319 (18)	0.35286 (18)	0.02730 (5)	0.0176 (4)	
C14	0.50604 (18)	0.3682 (2)	0.06095 (5)	0.0193 (4)	
H14A	0.5795	0.3125	0.0655	0.023*	
C15	0.46928 (18)	0.46577 (19)	0.08730 (5)	0.0180 (4)	
H15A	0.5176	0.4743	0.1096	0.022*	
C16	0.40164 (19)	0.2288 (2)	−0.03062 (5)	0.0207 (4)	
H16A	0.4010	0.3125	−0.0454	0.025*	
H16B	0.3101	0.2034	−0.0251	0.025*	
C17	0.47013 (19)	0.11601 (19)	−0.05233 (5)	0.0211 (4)	
H17A	0.4708	0.0334	−0.0371	0.025*	
H17B	0.5620	0.1420	−0.0571	0.025*	
C18	0.4013 (2)	0.0863 (2)	−0.08992 (5)	0.0229 (4)	
H18A	0.3874	0.1717	−0.1033	0.027*	
H18B	0.3145	0.0462	−0.0850	0.027*	
C19	0.48161 (19)	−0.0107 (2)	−0.11501 (5)	0.0219 (4)	
H19A	0.4997	−0.0939	−0.1009	0.026*	
H19B	0.5667	0.0318	−0.1208	0.026*	
C20	0.4129 (2)	−0.0490 (2)	−0.15221 (5)	0.0274 (5)	
H20A	0.3798	0.0336	−0.1642	0.033*	
H20B	0.4781	−0.0899	−0.1691	0.033*	
C21	0.2977 (2)	−0.1482 (2)	−0.14688 (6)	0.0286 (5)	
H21A	0.2614	−0.1722	−0.1711	0.043*	
H21B	0.2297	−0.1057	−0.1317	0.043*	
H21C	0.3291	−0.2292	−0.1344	0.043*	
H1O1	0.529 (3)	0.886 (3)	0.3205 (7)	0.057 (8)*	

### Atomic displacement parameters (Å^2^)

**Table:**

	*U*^11^	*U*^22^	*U*^33^	*U*^12^	*U*^13^	*U*^23^
O1	0.0221 (7)	0.0227 (7)	0.0162 (6)	0.0010 (6)	−0.0029 (6)	−0.0022 (6)
O2	0.0217 (7)	0.0263 (7)	0.0169 (6)	0.0043 (6)	−0.0023 (5)	−0.0003 (6)
O3	0.0243 (7)	0.0219 (7)	0.0160 (6)	0.0026 (6)	−0.0010 (5)	−0.0046 (5)
C1	0.0195 (9)	0.0155 (9)	0.0182 (9)	−0.0003 (8)	0.0009 (8)	−0.0010 (7)
C2	0.0206 (10)	0.0158 (9)	0.0184 (9)	0.0010 (8)	−0.0036 (8)	0.0011 (7)
C3	0.0191 (9)	0.0193 (9)	0.0132 (8)	−0.0041 (7)	−0.0003 (7)	0.0001 (7)
C4	0.0196 (9)	0.0177 (9)	0.0221 (9)	0.0013 (8)	0.0019 (8)	−0.0025 (8)
C5	0.0192 (9)	0.0193 (9)	0.0194 (9)	0.0008 (8)	−0.0023 (7)	−0.0002 (8)
C6	0.0179 (9)	0.0163 (8)	0.0148 (8)	−0.0011 (7)	0.0003 (7)	0.0014 (7)
C7	0.0199 (9)	0.0173 (9)	0.0153 (9)	−0.0027 (8)	0.0010 (7)	0.0023 (7)
C8	0.0172 (9)	0.0183 (9)	0.0175 (9)	0.0012 (8)	0.0004 (7)	0.0023 (7)
C9	0.0179 (9)	0.0194 (9)	0.0178 (9)	−0.0005 (7)	0.0020 (7)	0.0020 (8)
C10	0.0189 (9)	0.0177 (9)	0.0141 (8)	−0.0026 (7)	0.0012 (7)	0.0015 (7)
C11	0.0201 (10)	0.0217 (10)	0.0184 (9)	0.0016 (8)	−0.0006 (8)	0.0025 (8)
C12	0.0228 (10)	0.0239 (10)	0.0135 (8)	−0.0005 (8)	−0.0026 (8)	−0.0007 (8)
C13	0.0211 (9)	0.0161 (9)	0.0155 (9)	−0.0023 (7)	0.0032 (7)	0.0018 (7)
C14	0.0167 (9)	0.0218 (9)	0.0195 (9)	0.0001 (8)	0.0001 (7)	0.0021 (8)
C15	0.0198 (9)	0.0214 (9)	0.0128 (8)	−0.0032 (8)	0.0001 (7)	0.0017 (7)
C16	0.0235 (10)	0.0228 (10)	0.0157 (9)	−0.0004 (8)	−0.0013 (8)	0.0010 (8)
C17	0.0238 (10)	0.0208 (9)	0.0188 (9)	0.0022 (8)	0.0017 (8)	−0.0009 (8)
C18	0.0270 (11)	0.0223 (10)	0.0194 (9)	0.0038 (8)	0.0010 (8)	−0.0017 (8)
C19	0.0253 (10)	0.0203 (9)	0.0201 (9)	−0.0005 (8)	0.0050 (8)	−0.0014 (8)
C20	0.0399 (13)	0.0245 (10)	0.0178 (10)	−0.0012 (10)	0.0031 (9)	−0.0018 (8)
C21	0.0344 (12)	0.0286 (11)	0.0228 (10)	0.0021 (10)	−0.0038 (9)	−0.0021 (9)

### Geometric parameters (Å, °)

**Table:**

O1—C3	1.359 (2)	C12—C13	1.390 (3)
O1—H1O1	0.89 (3)	C12—H12A	0.9300
O2—C7	1.243 (2)	C13—C14	1.403 (2)
O3—C13	1.366 (2)	C14—C15	1.382 (3)
O3—C16	1.442 (2)	C14—H14A	0.9300
C1—C2	1.388 (2)	C15—H15A	0.9300
C1—C6	1.397 (2)	C16—C17	1.508 (3)
C1—H1A	0.9300	C16—H16A	0.9700
C2—C3	1.389 (2)	C16—H16B	0.9700
C2—H2A	0.9300	C17—C18	1.524 (3)
C3—C4	1.395 (3)	C17—H17A	0.9700
C4—C5	1.380 (2)	C17—H17B	0.9700
C4—H4A	0.9300	C18—C19	1.526 (3)
C5—C6	1.401 (2)	C18—H18A	0.9700
C5—H5A	0.9300	C18—H18B	0.9700
C6—C7	1.481 (2)	C19—C20	1.530 (3)
C7—C8	1.468 (3)	C19—H19A	0.9700
C8—C9	1.338 (2)	C19—H19B	0.9700
C8—H8A	0.9300	C20—C21	1.519 (3)
C9—C10	1.457 (2)	C20—H20A	0.9700
C9—H9A	0.9300	C20—H20B	0.9700
C10—C11	1.394 (2)	C21—H21A	0.9600
C10—C15	1.405 (3)	C21—H21B	0.9600
C11—C12	1.385 (3)	C21—H21C	0.9600
C11—H11A	0.9300		
			
C3—O1—H1O1	110.8 (17)	C15—C14—H14A	119.8
C13—O3—C16	117.35 (14)	C13—C14—H14A	119.8
C2—C1—C6	121.13 (17)	C14—C15—C10	120.75 (17)
C2—C1—H1A	119.4	C14—C15—H15A	119.6
C6—C1—H1A	119.4	C10—C15—H15A	119.6
C1—C2—C3	119.76 (17)	O3—C16—C17	107.68 (15)
C1—C2—H2A	120.1	O3—C16—H16A	110.2
C3—C2—H2A	120.1	C17—C16—H16A	110.2
O1—C3—C2	122.84 (16)	O3—C16—H16B	110.2
O1—C3—C4	117.15 (16)	C17—C16—H16B	110.2
C2—C3—C4	120.01 (16)	H16A—C16—H16B	108.5
C5—C4—C3	119.72 (17)	C16—C17—C18	112.12 (16)
C5—C4—H4A	120.1	C16—C17—H17A	109.2
C3—C4—H4A	120.1	C18—C17—H17A	109.2
C4—C5—C6	121.30 (17)	C16—C17—H17B	109.2
C4—C5—H5A	119.4	C18—C17—H17B	109.2
C6—C5—H5A	119.4	H17A—C17—H17B	107.9
C1—C6—C5	118.05 (16)	C17—C18—C19	112.69 (16)
C1—C6—C7	123.00 (16)	C17—C18—H18A	109.1
C5—C6—C7	118.90 (16)	C19—C18—H18A	109.1
O2—C7—C8	119.66 (16)	C17—C18—H18B	109.1
O2—C7—C6	119.61 (16)	C19—C18—H18B	109.1
C8—C7—C6	120.73 (16)	H18A—C18—H18B	107.8
C9—C8—C7	119.77 (17)	C18—C19—C20	114.40 (17)
C9—C8—H8A	120.1	C18—C19—H19A	108.7
C7—C8—H8A	120.1	C20—C19—H19A	108.7
C8—C9—C10	129.03 (18)	C18—C19—H19B	108.7
C8—C9—H9A	115.5	C20—C19—H19B	108.7
C10—C9—H9A	115.5	H19A—C19—H19B	107.6
C11—C10—C15	117.62 (17)	C21—C20—C19	113.07 (16)
C11—C10—C9	118.60 (17)	C21—C20—H20A	109.0
C15—C10—C9	123.68 (16)	C19—C20—H20A	109.0
C12—C11—C10	122.42 (18)	C21—C20—H20B	109.0
C12—C11—H11A	118.8	C19—C20—H20B	109.0
C10—C11—H11A	118.8	H20A—C20—H20B	107.8
C11—C12—C13	119.14 (17)	C20—C21—H21A	109.5
C11—C12—H12A	120.4	C20—C21—H21B	109.5
C13—C12—H12A	120.4	H21A—C21—H21B	109.5
O3—C13—C12	124.91 (16)	C20—C21—H21C	109.5
O3—C13—C14	115.42 (16)	H21A—C21—H21C	109.5
C12—C13—C14	119.66 (17)	H21B—C21—H21C	109.5
C15—C14—C13	120.37 (18)		
			
C6—C1—C2—C3	−1.6 (3)	C8—C9—C10—C15	0.9 (3)
C1—C2—C3—O1	−179.56 (16)	C15—C10—C11—C12	1.6 (3)
C1—C2—C3—C4	0.2 (3)	C9—C10—C11—C12	−174.97 (17)
O1—C3—C4—C5	−179.55 (16)	C10—C11—C12—C13	−0.8 (3)
C2—C3—C4—C5	0.7 (3)	C16—O3—C13—C12	0.8 (3)
C3—C4—C5—C6	−0.2 (3)	C16—O3—C13—C14	179.42 (15)
C2—C1—C6—C5	2.0 (3)	C11—C12—C13—O3	177.71 (17)
C2—C1—C6—C7	−175.46 (17)	C11—C12—C13—C14	−0.9 (3)
C4—C5—C6—C1	−1.1 (3)	O3—C13—C14—C15	−177.06 (16)
C4—C5—C6—C7	176.46 (17)	C12—C13—C14—C15	1.7 (3)
C1—C6—C7—O2	162.42 (17)	C13—C14—C15—C10	−0.8 (3)
C5—C6—C7—O2	−15.0 (3)	C11—C10—C15—C14	−0.8 (3)
C1—C6—C7—C8	−16.6 (3)	C9—C10—C15—C14	175.58 (17)
C5—C6—C7—C8	165.93 (16)	C13—O3—C16—C17	176.52 (15)
O2—C7—C8—C9	5.7 (3)	O3—C16—C17—C18	−179.69 (15)
C6—C7—C8—C9	−175.26 (17)	C16—C17—C18—C19	170.71 (16)
C7—C8—C9—C10	−174.51 (17)	C17—C18—C19—C20	177.08 (17)
C8—C9—C10—C11	177.22 (19)	C18—C19—C20—C21	−73.4 (2)

### Hydrogen-bond geometry (Å, °)

**Table:**

*D*—H···*A*	*D*—H	H···*A*	*D*···*A*	*D*—H···*A*
O1—H1O1···O2^i^	0.89 (3)	1.77 (3)	2.6466 (19)	169 (2)
C1—H1A···O1^ii^	0.93	2.55	3.458 (2)	164

Symmetry codes: (i) *x*+1/2, *y*, −*z*+1/2; (ii) −*x*+1, *y*−1/2, −*z*+1/2.
